# Effect of Hawthorn Powder on Physical, Functional, and Sensory Characteristics of Set-Type Yogurt

**DOI:** 10.3390/foods14081377

**Published:** 2025-04-16

**Authors:** Jingjing Wang, Zeyuan Kang, Lifei Tang, Wenpei Qiu, Yuxin Wang, Tao Zhang, Daodong Pan, Maolin Tu

**Affiliations:** 1 Zhejiang Key Laboratory of Food Microbiology and Nutritional Health, Zhejiang-Malaysia Joint Research Laboratory for Agricultural Product Processing and Nutrition, College of Food Science and Engineering, Ningbo University, Ningbo 315800, China; jingjingwang0422@163.com (J.W.); kangzeyuannbu@163.com (Z.K.); 18193132882@163.com (L.T.); qiuwenpei2000@163.com (W.Q.); 13750645190@163.com (Y.W.); zhangtao@nbu.edu.cn (T.Z.); daodongpan@163.com (D.P.); 2 State Key Laboratory for Managing Biotic and Chemical Threats to the Quality and Safety of Agro-Products, Ningbo University, Ningbo 315211, China

**Keywords:** hawthorn powder, yogurt, quality, antioxidant activity

## Abstract

Plant powders have exhibited great potential to enhance the antioxidant activity of yogurt. This study aims to evaluate the effects of hawthorn powder (1.0–3.0%, *w*/*w*) addition on the antioxidant activity and physical properties of set-type yogurt. The results demonstrated that yogurt containing 1–2% hawthorn powder exhibited improvements in quality, water-holding capacity, and texture. Notably, the antioxidant activities, including the DPPH radical, hydroxyl radical scavenging rate, and Fe^2+^ chelating activity, reached 68.2 ± 4.9%, 81.6 ± 0.5%, and 72.2 ± 2.0%, respectively, and were higher than those of ordinary yogurt. Microstructural observation revealed that appropriate hawthorn powder concentration promoted homogeneous protein network formation, contributing to improved texture stability. In conclusion, this research is of interest to the development of functional dairy products.

## 1. Introduction

Yogurt is one of the most popular dairy products. It is made from fresh milk fermented by lactic acid bacteria. Yogurt not only retains the nutritional value of the original dairy but also possesses various potential health benefits, such as alleviating gastrointestinal discomfort, improving immune function, and preventing colorectal cancer [1]. Additionally, yogurt possesses antioxidant properties [2]. It is highly favored by consumers and remains one of the most widely consumed fermented dairy products worldwide. Nevertheless, some studies have suggested that yogurt may not be effective in achieving improvements in specific bodily functions, including improving high-density lipoprotein cholesterol and triglyceride levels [3]. Meanwhile, with the evolving consumer preferences for healthy ingredients and their growing pursuit of new flavors and varieties of fermented dairy products, the development of novel functional health foods has been attracting increasing attention.

So far, a variety of natural products have been applied in the research and development of functional fermented yogurt, such as mung bean, menthol extract, and beetroot puree [4,5,6]. Hawthorn is rich in flavonoids, organic acids, terpenes, and other nutrients. It possesses antioxidant, anti-inflammatory, and anti-cardiovascular disease properties as well as biological activities such as immunomodulation [7]. Some research shows that hawthorn powder can improve the quality of products and increase the flavor of the food at the same time. It was shown that a mixture made by mixing hawthorn flour with corn starch and heating could reduce starch contact with water and delay starch pasting, thus improving starch properties [8].

During oxidative stress, intracellular free radicals and other reactive oxygen species (ROS) within organisms cause damage to biomolecules, including proteins, lipids, and DNA. This damage subsequently results in cellular injury, tissue aging, and the onset of various diseases. Effective mitigation of oxidative stress can be achieved by removing excess ROS from the body, thereby reducing the risk of diseases such as cardiovascular disorders [9]. Unfortunately, there is little evidence available regarding the basic properties and free radical scavenging ability of hawthorn powder in yogurt. Meanwhile, combined with the functional properties of hawthorn itself, we infer that, by adding hawthorn powder to yogurt, the active substances in hawthorn can still exert their powerful effects. Consequently, this research aimed to investigate how various hawthorn powder concentrations affected the flavor and microstructure of yogurt and to further investigate the textural and antioxidant activities. It is of great significance to the development and utilization of functional dairy products and also provides new ideas for studies on the application of hawthorn components in dairy products.

## 2. Materials and Methods

### 2.1. Materials

Pure hawthorn powder was purchased from Pingyi Yuminzhuang Food Co., Ltd. (Shandong, China). Whole milk powder was obtained from Fonterra Trading Co., Ltd. (Shanghai, China). Food-grade sugar was acquired from Guangzhou Fuzheng Donghai Food Co., Ltd. (Guangzhou, China). Yogurt fermenter [composed of *Lactobacillus bulgaricus* and *Streptococcus thermophilus* in the ratio of 1:1 (*w*/*w*)] was obtained from Angel Yeast Co., Ltd. (Yichang, China). 2,2-diphenyl-1-picrylhydrazyl (DPPH) and ferrous chloride-tetrahydrate were procured from Shanghai Macklin Biochemical Technology Co., Ltd. (Shanghai, China). Ferrozine was purchased from Beijing Solarbio Technology Co., Ltd. (Beijing, China). De Man, Rogosa and Sharpe (MRS) agar was obtained from Beijing Aoboxing Biology Technology Co., Ltd. (Beijing, China). All other reagents used were of analytical grade.

### 2.2. Yogurt Production

**Figure 1:**
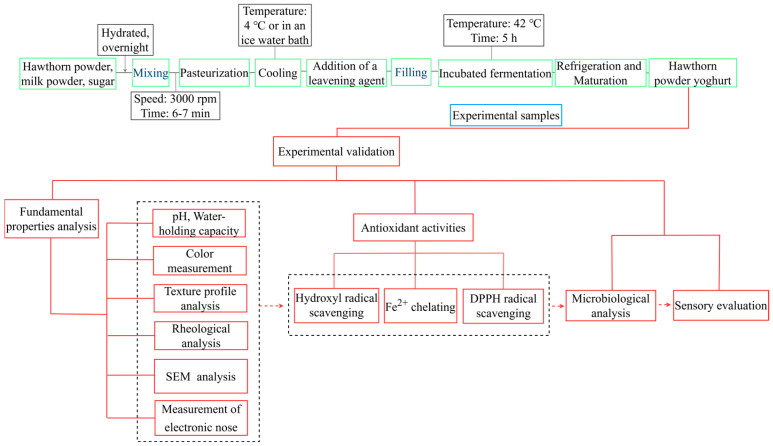
Flow chart of hawthorn powder yogurt production and activity determination. The principal processes involved in the production of yogurt, the fundamental parameters measured, and the primary indicators for the assessment of antioxidant activity were comprehensively delineated.

Firstly, 15% (*w*/*v*) whole milk powder and 5% (*w*/*v*) sugar were mixed in 100 mL of deionized water. Subsequently, 1.0%, 1.5%, 2.0%, 2.5%, and 3.0% (*w*/*v*) hawthorn powder were added to the mixture. It was magnetically stirred and hydrated overnight. The mixture was homogenized six to seven times for 30 s (3000 rpm/min) using a high-speed disperser (Ningbo Scientz Biotechnology Co., Ltd., Ningbo, China). After pasteurization and cooling to 42 °C, 0.3% (*w*/*v*) of yogurt fermenter was added for a fermentation period of 5 h at 42 °C. The fermentation was terminated when the yogurt had coagulated and the pH value decreased to 4.6. The samples were then kept at 4 °C for 24 h to ripen and stored at 4 °C. The control group consisted of samples without added hawthorn powder. After the hawthorn powder yogurt matured, samples were immediately collected for subsequent analysis of basic characteristics and antioxidant activity. The preparation process is shown in Figure 1.

### 2.3. Preparation of Aqueous Extract of Yogurt

The preparation of aqueous extracts of yogurt followed the method described by Shori et al. [10], with minor adjustments. Firstly, the yogurt sample was diluted with distilled water in a ratio of 1:0.25. The sample was homogenized for 10 s at 8000 rpm. The pH of the yogurt solution was adjusted to 4.0 with HCl (0.5 M) and incubated in a water bath at 45 °C for 10 min; then, the yogurt solution was centrifuged for 10 min (8000 rpm/min) to remove the milk proteins in the precipitate, and the supernatant was collected. After that, the pH of the above yogurt supernatant was adjusted to 7.0 by adding NaOH (0.5 M). The sample was centrifuged for 10 min (5000 rpm/min) to further precipitate the proteins. Finally, the yogurt supernatant was collected and lyophilized and stored in a refrigerator at −20 °C to be used for the determination of antioxidant activity.

### 2.4. Measurement of pH and Water-Holding Capacity

The pH of the yogurt with varying concentrations of hawthorn powder was tested using a pH meter (Five Easy Plus FE28, Mettler Toledo, Shanghai, China). The water-holding capacity (WHC) was determined based on the method described by Jia et al. [11], with modifications. Specifically, the method involves centrifugation of a 10 g sample of yogurt at 4 °C for 25 min (4000 rpm/min) to separate the whey from the sediment. The WHC was then calculated using the following Formula (1):

(1) $$WHC\;(\%)=\frac{Weight\;of\;sediment\;(g)}{Weight\;of\;yogurt\;(g)}\times 100$$

### 2.5. Color Measurement

Color was measured by using a colorimeter (CR-400, Konica Minolta, Tokyo, Japan). During the test, the sample was taken into a beaker and repeated three times at different positions. The values were read out directly on the colorimeter, where L* indicates the brightness value, a* indicates the red and green intensity value, and b* indicates the yellow and blue intensity value [12].

### 2.6. Rheological Analysis

The rheological properties of the yogurt were determined by a rheometer (DHR-2, Ta Instruments, New Castle, DE, USA) using the method of Pachekrepapol et al. [13], with some adjustments. The aim was to measure the effect of different additions of hawthorn powder on the energy storage modulus G′ and loss modulus G″ of the yogurt samples subjected to frequency scanning. The measurements were carried out using a rheometer with a concentric cylinder with a 40 mm solid stator rotor, and a small amount of stirred fresh yogurt samples were taken and placed on the rheometer under the following conditions: a gap of 1 mm, a temperature of 25 °C, a scanning frequency of 0.1 to 10 Hz, and a strain of 0.75%.

### 2.7. Texture Profile Analysis (TPA)

The analysis of the texture properties of the yogurt samples was performed in a texture analyzer (TA.XTplus, Stable Micro Systems, Godalming, Surrey, UK). The experimental protocol was modified from the method described in previous research [14]. The texture characteristics of the hawthorn powder yogurt were measured using a physical property analyzer with the TPA mode. The pre-measurement rate was 2.0 mm/s, the mid-measurement rate was 1.0 mm/s, and the post-measurement rate was 2.0 mm/s. The downward pressure interval was 1 s, and the minimum trigger force was 0.3 N. The measurements were taken using a cylindrical probe with a diameter of 36 mm (P36R). The hardness, chewiness, viscosity, and cohesion of the samples were recorded.

### 2.8. Scanning Electron Microscopy (SEM) Analysis

The microstructure of the hawthorn powder yogurt was observed using a scanning electron microscope (Hitachi S-3400N, Tokyo, Japan). That is, a small amount of hawthorn powder yogurt sample was taken on the inner wall of a Petri dish, coated evenly, placed in a −20 °C refrigerator for pre-freezing, and then quickly put into a vacuum freeze dryer for the drying process. Then, the sample was plated with gold by an ion sputtering method, and the microstructure of the yogurt sample could be observed by scanning electron microscope filming, scanning, and observing the sample under the electron accelerating voltage of 10 kV and the magnification of 2000×.

### 2.9. Measurement of Electronic Nose

Flavor determination of the yogurt samples was performed using an electronic nose (PEN3, AIRSENSE, Schwerin, Germany). The experimental setup followed the approach described by Li et al. [15], with some adjustments. A 5 g yogurt sample was placed in a 20 mL glass bottle and sealed and left to stand at 25 °C for 30 min. The electronic nose data acquisition time was 100 s. The carrier gas was dry clean air with an internal flow rate of 400 mL/min, the inlet flow rate was 400 mL/min, the sample preparation time was 5 s, and the sensor cleaning time was 60 s. The measurements were repeated three times for each sample, and the average value was taken. The performance of the electronic nose sensor is described in Table 1.

### 2.10. Antioxidant Properties

The DPPH radical scavenging activity assay was carried out according to Wang et al. [16], with minor adjustments. The freeze-dried hawthorn powder yogurt aqueous extract was dissolved in deionized water (15 mg/mL), DPPH was dissolved in ethanol (0.2 mM), and then equal volumes of the sample solution and DPPH were mixed thoroughly, and the reaction was carried out at 37 °C under dark conditions for 30 min. The absorbance value was measured at 517 nm, deionized water was used instead of the sample solution as a blank group, and three parallels were made for each sample. The percentage of DPPH radical inhibition by yogurt was calculated according to Equation (2):

(2) $$DPPH\;radical\;scavenging\;rate\;(\%)=\frac{\left(A_{0}-A_{1}\right)}{A_{0}}\times 100$$

The modified method was used to determine hydroxyl radical scavenging activity of the yogurt samples [17]. The aqueous extract of freeze-dried hawthorn powder yogurt was dissolved in deionized water (15 mg/mL), and an equal volume of the sample solution, FeSO_4_ (9 mM), and H_2_O_2_ (8.8 mM) were mixed thoroughly. The reaction was carried out at 37 °C for 10 min in darkness, and then an equal volume of salicylic acid-ethanol solution (9 mM) was added to the sample solution. The reaction was carried out at 37 °C for 30 min in darkness. The absorbance value was measured at 510 nm. The absorbance value was determined at 510 nm; deionized water was used as a blank group instead of the sample solution, and three parallels were made for each sample. The percentage inhibition of hydroxyl radicals by yogurt was calculated according to Equation (3):

(3) $$Hydroxyl\;radical\;scavenging\;rate\;(\%)=\frac{\left(A_{0}-A_{1}\right)}{A_{0}}\times 100$$

Fe^2+^ chelating activity was assessed based on the procedure outlined by Wang et al. [18], with small modifications. The aqueous extract of freeze-dried hawthorn powder yogurt was dissolved in deionized water (15 mg/mL). The sample solution (600 μL) was taken and mixed with FeCl_2_ solution (10 μL, 2 mM, dissolved in deionized water) and Ferrozine solution (20 μL, 5 mM, dissolved in deionized water) and then incubated at room temperature under dark conditions for 10 min. The absorbance value was measured at 562 nm, and deionized water was used instead of the sample solution. The absorbance values were determined at 562 nm, deionized water was used as the blank group, and 3 parallels were made for each sample. Calculation of Fe^2+^ chelating activity was calculated according to Equation (4):

(4) $${Fe}^{2+}chelating\;activity\;(\%)=\frac{\left(A_{0}-A_{1}\right)}{A_{0}}\times 100$$

In the above equation, $A_{1}$ indicates the absorbance value of the sample group, and $A_{0}$ indicates the absorbance value of the blank group.

### 2.11. Assessment of Lactic Acid Bacteria Viability

The total viable counts of lactic acid bacteria (LAB) were assessed in accordance with the Chinese National Food Safety Standard (GB 4789.35-2023) [19] following a 24 h storage period at 4 °C. The yogurt samples were subjected to serial dilutions and plated on MRS agar, followed by incubation at 37 °C for 48 h to facilitate enumeration. The results were expressed as log CFU/mL.

### 2.12. Sensory Evaluation of Hawthorn Powder Yogurt

The sensory assessment was conducted by 20 individuals who had received professional training in this field. All participants voluntarily engaged in the experiment. In this study, the privacy rights of all participants were fully protected, and they had the right to unconditionally withdraw from the experiment at any time. The evaluation includes color, tissue state, flavor, and texture, with a score of 100 points, and the scoring criteria are shown in Table 2. Before each tasting session, all group members thoroughly rinsed their mouths to ensure that no residual substances interfered with the assessment.

### 2.13. Data Processing and Analysis

All measurements in this work were conducted at least three times independently. The experimental data were visualized using the GraphPad Prism software (v10.3.0, GraphPad Software, San Diego, CA, USA) and the Origin 2021 (OriginLab Corp., Northampton, MA, USA) software. Statistical significance (*p* < 0.05) was analyzed using a one-way analysis of variance followed by Duncan’s test, and the results were expressed as the mean ± standard error of the mean (SEM). Statistical analyses were performed using the GraphPad Prism software.

## 3. Results and Discussion

### 3.1. Effect of Hawthorn Powder on pH and Water-Holding Capacity of Yogurt

**Figure 2:**
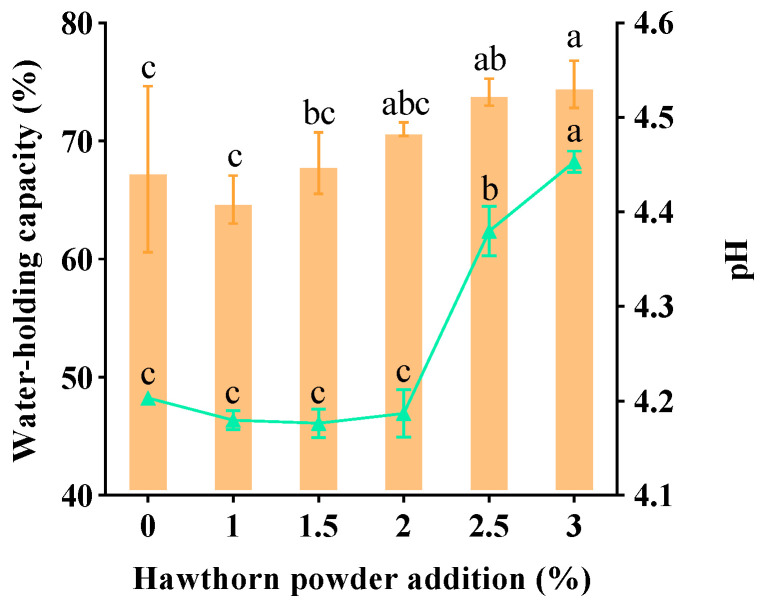
The effect of hawthorn powder addition on the water-holding capacity and pH value of yogurt. Different letters (a, b, c) in the figure indicate statistically significant differences between groups (*p* < 0.05).

WHC is among the most significant factors in determining the quality of yogurt and can be used as an indicator of the quality of fermented dairy products [20]. Yogurt is rich in casein, and its protein molecules can build a reticulation structure between them, a structure that can hold water and other small molecules. Figure 2 demonstrates that the WHC of the yogurt increased gradually with the addition of hawthorn powder. When the additional amount of hawthorn powder is 3%, the WHC of the yogurt reaches the highest value, and the additional amount of hawthorn powder can significantly increase the WHC of the yogurt, which may be because hawthorn is rich in pectin, which confines the water molecules in the protein mesh structure [21]. The final solidification of the emulsion to form yogurt is related to the pH in the system. During yogurt fermentation, lactic acid is produced by the fermentation of lactic acid bacteria, and the accumulation of lactic acid leads to a decrease in the pH of the system until it reaches the isoelectric point of casein, which is 4.6 or lower. At this point, the casein micelles aggregate, and curdled yogurt is formed [22]. With the increase in hawthorn powder addition, the pH value of the hawthorn powder yogurt first decreases and then increases (Figure 2). The decrease in pH may be due to the lactic acid produced by *Lactobacillus bulgaricus* and *Streptococcus thermophilus* [23]. The pH value of the hawthorn powder yogurt ranged from 4.2 to 4.5. The yogurt samples containing 2.5% and 3.0% hawthorn powder had significantly higher pH values than the control. This could be due to the addition of excessive hawthorn powder, which contains hawthorn polysaccharides and phenolics that may have delayed the peak of acid production by the lactic acid bacteria, thus reducing the amount of acid produced.

### 3.2. Effect of Hawthorn Powder on the Color of Yogurt

The color of the yogurt changed significantly with an increase in the addition of hawthorn powder (Table 3). The color measurement results showed that the a* and b* values of the samples remarkably increased (*p* < 0.05), while the L* value gradually decreased (*p* < 0.05), indicating that the color of the yogurt gradually shifted towards red-yellow, accompanied by a decrease in brightness. The likely reason for these changes is the presence of natural pigments, such as anthocyanins, in hawthorn [24]. Specifically, with increasing concentrations of hawthorn powder, the pigment concentration elevated, which was incorporated into the yogurt matrix, thereby altering the surface color of the hawthorn powder yogurt.

### 3.3. Effect of Hawthorn Powder on the Rheological Properties of Yogurt

**Figure 3:**
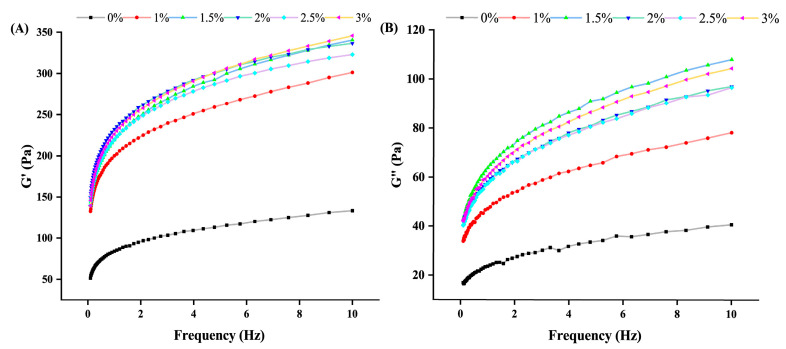
Rheological properties of hawthorn powder yogurt prepared at different concentrations (0%, 1%, 1.5%, 2%, 2.5%, 3%). (**A**,**B**) Frequency scanning tests. G′ and G″ represent the storage modulus and loss modulus, respectively.

Determination of the rheological properties of yogurt can be used to predict and control the final quality and structure of yogurt [25]. For viscoelastic materials, the energy storage modulus (G′) and loss modulus (G″) can describe the solid and elastic characteristics of the sample, respectively. Meanwhile, it can be seen from this experiment that the G′ values of the yogurt are much higher than the respective G″ values. Figure 3 shows that, with the gradual increase in hawthorn powder addition, G′ showed a trend of a gradual increase, and the change curve was higher than that of the control group. Among them, the G′ of all yogurt samples was significantly higher than G″ and increased with increasing frequency, a typical feature indicating the formation of a weak gel network with viscoelasticity in fermented milk. Hawthorn powder contains pectin, which, when added to yogurt under acidic conditions, can form a high-strength gel, significantly enhancing yogurt’s storage modulus. This is consistent with Linares-García et al. [26]. Therefore, this study indicates that the addition of appropriate hawthorn powder can effectively improve the flavor and quality of yogurt.

### 3.4. Effect of Hawthorn Powder on the Texture of Yogurt

**Figure 4:**
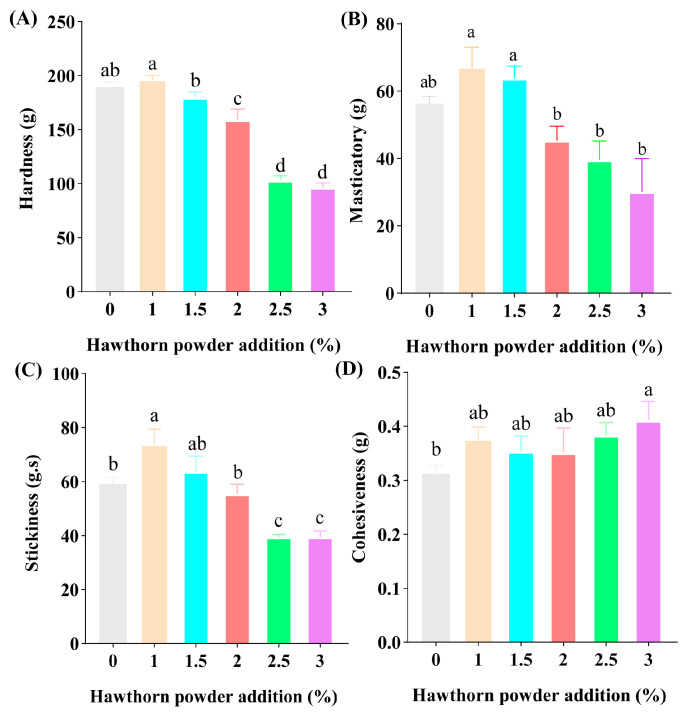
Effects of different additions of hawthorn powder on the texture of yogurt. (**A**) Hardness. (**B**) Masticatory. (**C**) Stickiness. (**D**) Cohesiveness. Different letters indicate statistically significant differences (*p* < 0.05).

The textural characteristics of yogurt, such as hardness and cohesion, are essential attributes for the quality and sensory evaluation of yogurt [27]. Figure 4 demonstrates that, when the addition of hawthorn powder was 1%, the hardness, masticatory, stickiness, and cohesiveness of the yogurt were remarkably enhanced (*p* < 0.05), which may be related to the presence of pectin and polyphenolic compounds in hawthorn powder. An appropriate amount of polyphenols and fibers in plant additives may promote protein cross-linking, while excessive amounts may lead to structural disruption [28]. Similarly, in the study on pomegranate peel extract-fortified yogurt, it was found that an addition of 0.5% significantly improved the hardness and WHC of the yogurt, while excessive addition (0.75%) led to a decrease in texture parameters [29]. However, as the hawthorn powder concentration rose to 2.0%, the hardness, masticatory, and stickiness values decreased. Notably, there may be an optimal threshold for hawthorn powder addition, beyond this threshold, excess fiber or polyphenols could disrupt the three-dimensional network structure of milk proteins, thereby altering the texture properties in yogurt.

### 3.5. Scanning Electron Microscopy Analysis of Hawthorn Powder Yogurt

**Figure 5:**
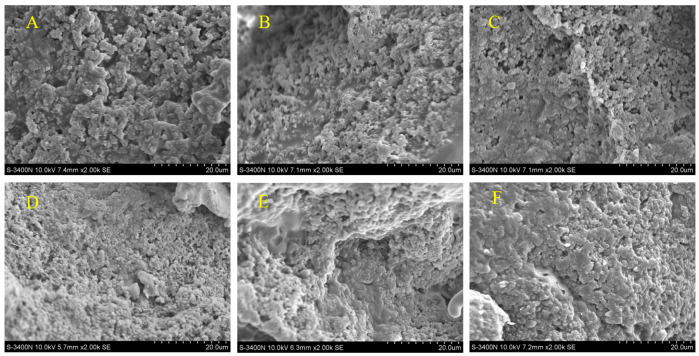
Scanning electron microscope images of yogurt samples with different concentrations of added hawthorn powder (2000×). (**A**) Control group. (**B**) 1% hawthorn powder. (**C**) 1.5% hawthorn powder. (**D**) 2% hawthorn powder. (**E**) 2.5% hawthorn powder. (**F**) 3% hawthorn powder.

Destruction of the microstructure of food produced during processing may affect the digestion and absorption of certain nutrients in the human gut [30]. The scanning electron microscopy results of yogurt samples with different additions of hawthorn powder are shown in Figure 5. It can be observed that the gel network of the control group sample is relatively sparse, while the microstructure undergoes certain changes when hawthorn powder is added. This may be due to the interaction between pectin and casein micelles contained in hawthorn powder. The pectin in hawthorn powder may embed into the casein network, reducing the gaps between protein clusters, thereby rendering the yogurt protein network more compact and stabilizing the gel structure of the yogurt [31]. Adding more than 1.5% of hawthorn powder causes the yogurt to become progressively softer. However, the microstructure demonstrated that the yogurt with hawthorn powder had a denser pore network than the control. This phenomenon may be related to the gelling properties of pectin in the hawthorn powder. Similar studies have indicated that the addition of plant-based ingredients, such as lentil flour, can modify the gel structure through interactions between starch or proteins and the milk matrix [32]. Furthermore, the pectin in hawthorn powder may fill the pores by forming a dense colloidal network (Figure 5) while simultaneously imparting higher elasticity to the gel, thereby reducing its hardness. This contrasts with the mechanism of starch absorption and stabilization of the structure in lentil flour, highlighting the differentiated regulatory effects of various functional ingredients on the texture of yogurt.

### 3.6. Hawthorn Powder Yogurt E-Nose Analysis

**Figure 6:**
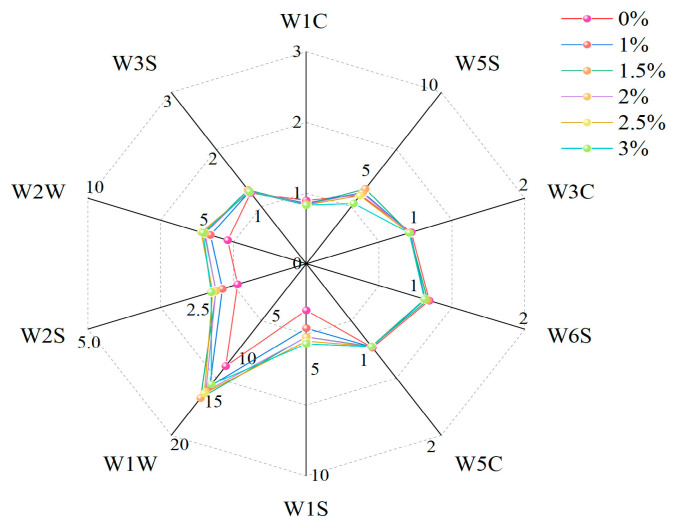
Electronic nose radargram of yogurt samples.

The e-nose is a valuable tool for rapidly evaluating volatile flavor compounds in fermented dairy products, where small changes in the sample can lead to differences in sensor response values [33]. The added hawthorn powder affects the flavor of yogurt to a certain extent. Among the 10 sensors, the response strengths of W1W, W2W, and W1S to the yogurt increased after the addition of hawthorn powder (Figure 6). These sensors were sensitive to sulfurous compounds, aromatic components, and methyl groups, indicating that the addition of hawthorn powder increased the levels of sulfurous compounds and aromatic substances such as terpenes. Hawthorn powdered yogurt undergoes oxidation or microbial action during storage and may generate terpene derivatives, the changes in which can be captured by the electronic nose via sensor W1W [34]. The low response values of the W1C and W3C sensors indicate that the addition of hawthorn powder enables the yogurt to remove some of the undesirable gaseous components.

### 3.7. Effect of Hawthorn Powder on Antioxidant Activity of Yogurt

Oxidative stress in an organism is a biochemical process that disrupts the redox balance due to excess intracellular oxidants [35]. Free radicals, on the other hand, are reactive oxygen species in cells that are constantly produced in the body and are capable of causing lipid peroxidation and protein damage [36]. Therefore, the antioxidant capacity of a sample can be determined by its free radical scavenging rate. In general, antioxidant capacity is influenced by complex factors with multiple mechanisms of action, and at least two methods are required to assess antioxidant activity in foods [37]. Additionally, polyphenols demonstrate strong antioxidant activity due to possessing hydroxyl substituent and aromatic structures [38]. And hawthorn contains polyphenolic compounds among its active ingredients, thus it is a good antioxidant [39]. Meanwhile, some research suggests that yogurt may be acting as a vehicle for delivering phenolic compounds [40]. Therefore, DPPH, hydroxyl radical scavenging assay, and Fe^2+^ chelating activity assay were performed to further investigate the antioxidant activity of the hawthorn powder yogurt.

**Figure 7:**
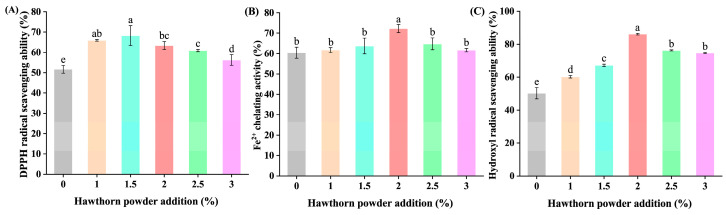
Effect of different hawthorn powder concentrations on the antioxidant activity of yogurt. (**A**) DPPH radical scavenging ability. (**B**) Fe^2+^ chelating activity. (**C**) Hydroxyl radical scavenging ability. The different letters indicate that the difference was statistically significant (*p* < 0.05).

The DPPH radical scavenging ability of the yogurt showed a tendency of first increasing and then decreasing with the increasing amount of hawthorn powder (Figure 7). Compared with the DPPH radical scavenging rate of 51.7% in the control yogurt, the scavenging rate reached up to 68.2% when the addition amount of hawthorn powder was 1.5%, and the DPPH radical scavenging rate of each sample group was obviously higher than that of the control group (*p* < 0.05). This might be caused by the ability of the polyphenols in hawthorn powder to inhibit lipid oxidation through hydrogen atoms or electron transfer mechanisms [41]. The antioxidant effect of the yogurt water extract of hawthorn powder was greatly increased. Meanwhile, the hydroxyl radical scavenging ability and Fe^2+^ chelating activity also showed a tendency to increase and then decrease (Figure 7).

This study found that the antioxidant activity of the yogurt was clearly influenced by the incorporation of hawthorn powder. As the amount of hawthorn powder increases, its activity decreases after reaching a certain threshold. Comparable discoveries were reported by Li et al. [42], who suggested that high concentrations of phenolic compounds might reduce activity due to limitations in solubility or oxidative polymerization. In addition, when hawthorn complexes were added to stirred yogurt, it was observed that excessive fiber (>0.5%) disrupted the gel network and increased the syneresis rate, indirectly accelerating the degradation of phenolic compounds [43]. This study suggests that a 2% addition level may represent the critical threshold for hawthorn powder in the yogurt system. Beyond this concentration, interactions between phenolics and casein, such as hydrogen bonding, may mask the antioxidant active sites, while particle aggregation leads to structural instability.

### 3.8. Analysis of Lactic Acid Bacteria Survival in Yogurt Samples

**Figure 8:**
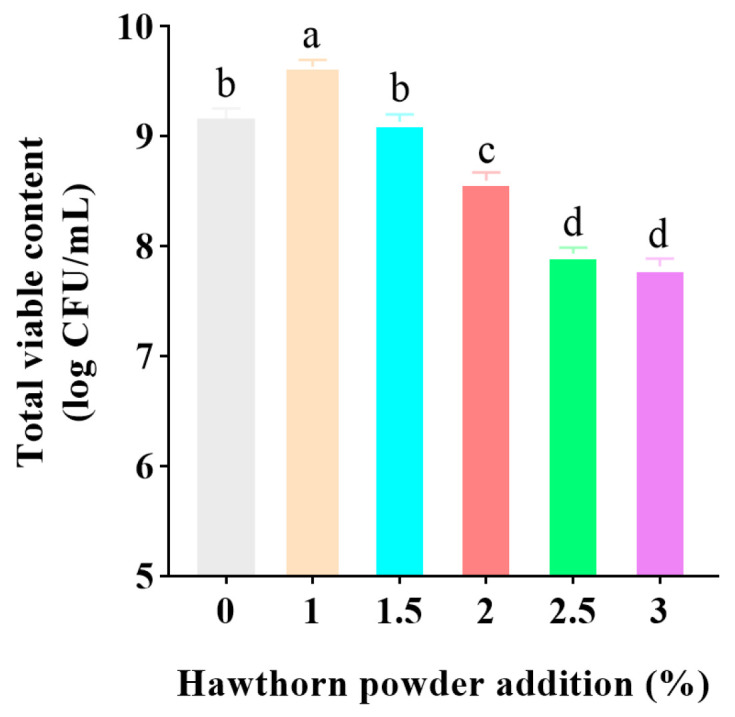
Effect of adding different amounts of hawthorn powder on the activity of lactic acid bacteria in yogurt. Different letters indicate statistically significant differences (*p* < 0.05).

The results of the microbial analysis showed that the LAB count in all yogurt samples remained above 7 log CFU/mL, in compliance with the minimum standard established by the Codex Alimentarius Commission (2022) [44] (Figure 8). It was observed that the lactic acid bacteria activity in all samples initially increased and then continuously declined. It was determined that the incorporation of 1% hawthorn powder led to an obvious enhancement in the proliferation of lactic acid bacteria. This effect may be attributable to the polyphenolic compounds present in hawthorn, which have been observed to promote bacterial growth by scavenging free radicals and mitigating oxidative stress. When the addition of hawthorn powder exceeded 1.5%, the survival rate of lactic acid bacteria gradually reduced. Excessive hawthorn powder may interfere with the formation of casein gels, leading to a looser texture and weakening the physical protective barrier of the strains, thereby hindering bacterial adhesion and growth. Similarly, the addition of an appropriate amount of potato powder in yogurt can promote the growth of lactic acid bacteria. However, excessive addition leads to a deterioration in the texture and flavor of the yogurt, with a corresponding decrease in the survival rate of lactic acid bacteria [45].

### 3.9. Effect of Hawthorn Powder on the Sensory Quality of Yogurt

**Figure 9:**
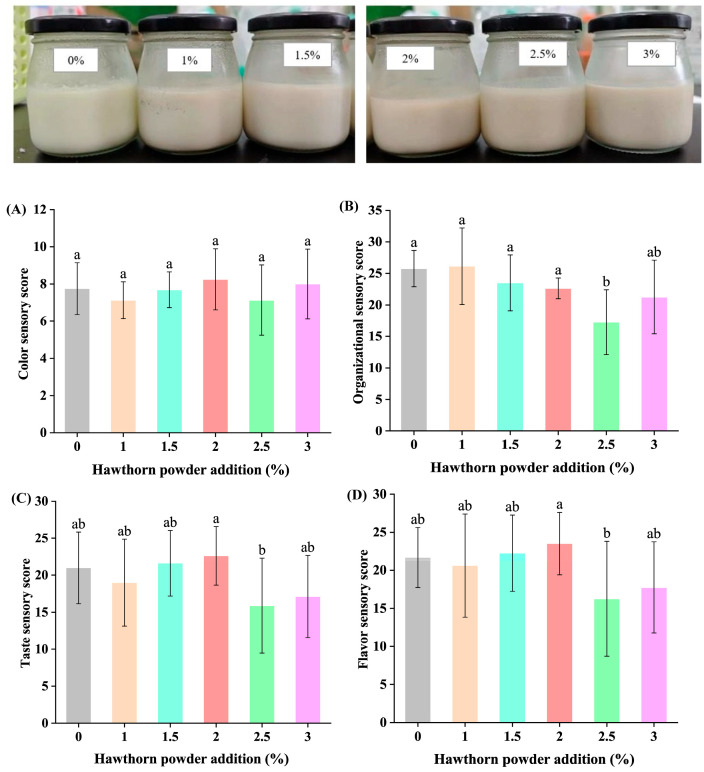
Visual appearance and sensory scores of yogurt. (**A**) Color sensory score. (**B**) Organizational sensory score. (**C**) Taste sensory score. (**D**) Flavor sensory score. Different letters indicate statistically significant differences (*p* < 0.05).

The hawthorn powdered yogurt product diagram is shown in Figure 9. When adding hawthorn powder from 1.5% to 2%, the sensory scores of the yogurt showed an increasing trend with increasing added amount (Figure 9). When hawthorn powder was added at 2%, the sensory scores of the yogurt reached the greatest value. This showed that hawthorn powder had a positive effect on improving the color, tissue state, flavor, and texture of the yogurt. However, when the addition level exceeded 2%, reaching 2.5%, the overall sensory scores began to decrease and were clearly less than in the control group (*p* < 0.05). Excessive hawthorn powder can adversely affect the final sensory score of yogurt. This may be due to the overuse of hawthorn powder, which partially disrupts the gel structure of the yogurt, leading to excessive whey separation and an elevated acidity, thereby impacting the sensory quality of the yogurt. Therefore, it can be inferred that there may be a threshold for the addition of hawthorn powder, beyond which the flavor and characteristics of the product are negatively affected.

## 4. Conclusions

In this study, the basic properties of yogurt with the addition of hawthorn powder were determined as well as the microstructure and antioxidant activity. The results demonstrated that moderate amounts of hawthorn powder could enhance the water-holding capacity, energy storage modulus, loss modulus, textural properties, and antioxidant activity of yogurt and improve its color. Specifically, the addition of 1% hawthorn powder resulted in the highest values for hardness, viscosity, and chewiness. Meanwhile, when hawthorn powder was added at concentrations ranging from 1.5% to 2%, the overall organoleptic scores were higher, and the DPPH radical scavenging, hydroxyl radical scavenging, and Fe^2^⁺ chelation activities exhibited significant increases, indicating a marked improvement in antioxidant capacity. The findings of this study provide new insights and potential strategies for developing and utilizing hawthorn-based processed products and fermented dairy products. In conclusion, it can be inferred that adding hawthorn powder effectively improves the structural properties, flavor, and overall quality of yogurt.

## Figures and Tables

**Table 1:** Array of 10 types of air-sensitive sensors for electronic noses and their characterization.

Serial Number	Transducers Performance Description	Performance Description
1	W1C	Sensitivity to aromatic ingredients
2	W5S	High sensitivity, sensitive to nitrogen oxides
3	W3C	Sensitive to aromatic ingredients, ammonia
4	W6S	Mainly sensitive to hydrogens
5	W5C	Hypoallergenic to short-chain alkanes, aromatic components
6	W1S	Sensitive to hydrocarbons, sensitive to methyl groups
7	W1W	Sensitive to sulfur compounds, terpenes, and sulfur organic compounds
8	W2S	Sensitive to aldehydes, alcohols, and ketones
9	W2W	Sensitive to aromatic components, organic sulfides
10	W3S	Sensitive to alkanes, methane

**Table 2:** Sensory scoring criteria for yogurt samples.

Projects	Scoring Standard	Total Points
Color	Uniform and consistent color, with the characteristic light reddish-brown of hawthorn yogurt (7–10)	10
	Varying shades of color, reddish-brown is lighter (3–6)	
	The color is uneven and does not have the characteristic light reddish-brown color of hawthorn yogurt (1–2)	
Organizational state	Good coagulation, no or little whey precipitation, no or little flocculation, uniform texture (25–30)	30
	Good coagulation, slight whey precipitation, small amounts of flocculent, layered (15–24)	
	Poor coagulation, large amounts of whey precipitated, more flocculent, with large granular clots (0–14)	
Flavor	A pronounced hawthorn yogurt flavor, with a harmonious fermented milky and hawthorn aroma (25–30)	30
	Light hawthorn yogurt flavor, with a harmonious fermented milky and hawthorn aroma (15–24)	
	Obvious hawthorn yogurt flavor, with a harmonious fermented milky and hawthorn aroma (0–14)	
Taste	Moderately sweet and sour, strong palatability, even curd, fine texture, unique hawthorn flavor (25–30)	30
	Average sweet and sour, more palatable, average curd, no graininess, weak hawthorn flavor (15–24)	
	Sour–sweet disharmony, unpleasant to the mouth, poor curd, poor taste, no hawthorn unique flavor (0–14)	

**Table 3:** Effect of hawthorn powder on the color of yogurt.

Additive Quantity (%)	L*	a*	b*
0	75.62 ± 0.83 ^ab^	−1.60 ± 0.06 ^c^	9.11 ± 0.51 ^b^
1.0	75.98 ± 1.59 ^a^	−0.18 ± 0.40 ^b^	9.63 ± 0.02 ^b^
1.5	71.91 ± 0.76 ^c^	0.22 ± 0.07 ^b^	9.52 ± 0.26 ^b^
2.0	72.92 ± 1.58 ^bc^	0.38 ± 0.47 ^b^	9.98 ± 0.64 ^b^
2.5	70.55 ± 1.05 ^cd^	1.18 ± 0.56 ^a^	11.53 ± 1.49 ^ab^
3.0	68.85 ± 2.48 ^d^	1.65 ± 0.11 ^a^	12.72 ± 0.40 ^a^

Note: Different letters indicate significant differences (*p* < 0.05). Comparisons with the control group show the significant differences.

## Data Availability

The original contributions presented in this study are included in the article. Further inquiries can be directed to the corresponding author.

## Abbreviations

The following abbreviations are used in this manuscript:

ROS	Reactive oxygen species
WHC	Water-holding capacity
TPA	Texture profile analysis
SEM	Scanning electron microscopy
DPPH	2,2-Diphenyl-1-picrylhydrazyl
